# Dynamics of psychological responses to COVID-19 in India: A longitudinal study

**DOI:** 10.1371/journal.pone.0240650

**Published:** 2020-10-13

**Authors:** Anvita Gopal, Anupam Joya Sharma, Malavika Ambale Subramanyam

**Affiliations:** 1 Cognitive Science, Indian Institute of Technology Gandhinagar, Gujarat, India; 2 Social Epidemiology, Indian Institute of Technology Gandhinagar, Gujarat, India; Ryerson University, CANADA

## Abstract

The novel COVID-19 pandemic has created chaos around the globe. To curb its spread, the Government of India announced a nationwide lockdown on March 24th, 2020 for 21 days, which was extended further for a longer time. This long period of lockdown disrupted the routine of all citizens, affecting their psychological well-being. While recent studies showed the psychological burden of Indians during the pandemic, no study has assessed whether the psychological toll changed over time due to repeated extensions of the lockdown. We followed up 159 Indian adults during the first two months of the lockdown to assess any change in their anxiety, stress, and depressive symptoms. Multilevel linear regression models of repeated observations nested within individuals adjusted for sociodemographic covariates showed that anxiety (**β** = 0.81, 95% CI: 0.03, 1.60), stress (**β** = 0.51, CI: 0.32, 0.70), and depressive symptoms (**β** = 0.37, CI: 0.13, 0.60) increased over time during the lockdown. This increase was higher among women than men independent of covariates. Individual resilience was negatively associated with adverse psychological outcomes. Our findings suggested that while the lockdown may help in effectively addressing this pandemic, the state and society at large need to be sensitive to the mental health impacts of a long-drawn-out lockdown. Such effects likely have long-term sequelae. The disproportionate impact on women needs immediate attention. Moreover, it behooves society to address the root causes driving the unequal distribution of psychological distress during such crises.

## Introduction

The novel coronavirus disease (COVID-19), which originated in China, was declared a public health emergency by the World Health Organization (WHO) on January 30th, 2020 [1]. After a steep global increase in the number of infected persons, different countries took various stringent measures to curb its spread, a nationwide “lockdown” was one such step. The Government of India called for a nationwide lockdown from March 25th, 2020 [2]. Citizens were mandated to stay at home, and all major offices, malls, factories, and schools were shut down for 21 days [2]. The lockdown was further extended until May 3rd, with conditional relaxations [3]. While the lockdown was intended to curb the spread of the virus, it had psychological ramifications on the citizens [4–7]. The self-isolation and restrictions on physical mobility due to the lockdown caused major disruptions to routines in life and hindered the ability to meet regular responsibilities—potentially affecting the physical and mental health of individuals. Recent studies have reported higher levels of stress [8, 9], anxiety [8, 10, 11], depression [10, 12, 13], and poor quality of life [9, 10] during the COVID-19 crisis in different populations. However, the repeated extensions in the lockdown period in India led to longer restrictions on physical mobility and prolonged self-isolation measures. This could have increased the intensity of negative psychological outcomes among Indians, leading to a poorer quality of life not just during the lockdown but also after the crisis. Previous studies have shown that prolonged periods of isolation and limited mobility significantly impact mental well-being [14, 15] during crises. Further, a prolonged experience of negative mental health outcomes could have adverse effects on physical health outcomes such as sleep disorders [16] and health-related quality of life [17].

While mental health effects due to the lockdown are likely to be seen among a majority of Indian adults, the impact of a lockdown *extended over longer periods* might differ across vulnerable groups [18]. For instance, the stress experienced by persons with limited adaptive capacity, fewer financial resources, lower social support, and pre-existing mental health conditions might be higher than among those who do not share these characteristics. With longer lockdown periods, financially weaker individuals might face more challenges in meeting the basic needs of their families. Moreover, the continued restrictions on physical mobility could place a higher burden on the social networks of vulnerable individuals, thus reducing their access to social support over time and impacting their adaptability. Additionally, in a patriarchal society such as India, with a high prevalence of domestic abuse [19], the lockdown [20] (especially limited mobility) can potentially increase the risk of experiencing domestic abuse. Prolonged exposure to the threat of domestic violence could worsen the mental health of women during this crisis.

Despite these risks, several resources that help with coping could be available to individuals. Previous studies have highlighted the role of social support in reducing anxiety and stress [21, 22]. Recent studies, focused on COVID-19 also support this [11, 23]. In addition to social support, there could also be several individual-level resources such as resilience that could help individuals face adversity [24]. Resilience helps to strengthen mental health and reduce the possibility of developing psychiatric morbidities, especially during the COVID-19 pandemic [25, 26]. There is scant research on the mental health effects of such protective factors during the extended lockdown in India.

Reliably measuring the impact of lockdowns that extend over a long period of time requires a longitudinal study design. While several cross-sectional studies [5–7] have focused on psychological well-being during COVID-19 in India, we could not locate any study investigating the *change* in such psychological outcomes during the lockdown period. A longitudinal investigation helps establish temporal sequence and document trends while exploring how the adverse psychological outcomes change (if at all) over time during the lockdown. To address this gap in the literature, we conducted a longitudinal study in four phases to investigate the changes in three psychological outcomes viz., anxiety, stress, and depressive symptoms, during the lockdown in India. The following were the aims of our study and our hypotheses:

1. The major aim of our study was to investigate how the levels of anxiety, stress, and depressive symptoms changed during the lockdown among Indian adults, independent of their age, gender, income, educational qualification, place of residence, and history of mental health. We hypothesized that as the period of lockdown increases, the levels of anxiety, stress, and depressive symptoms would also increase over time independent of the covariates.
2. The second aim was to find out whether the changes, if any, in the levels of anxiety, stress, and depressive symptoms, differed across gender. Based on the large body of research highlighting gender disparities in the risk of anxiety, stress, and depression [27], we hypothesized that in a patriarchal society such as India, compared to men, women will have a greater increase in levels of anxiety, stress, and depressive symptoms during the lockdown.
3. Thirdly, we assessed whether the protective factors, social support and resilience, were related to the participants’ anxiety, stress, and depressive symptoms during the lockdown. We hypothesized that greater social support and higher individual resilience will be related to lower levels of anxiety, stress, and depressive symptoms, independent of all covariates.

## Methods

### Recruitment of participants

We collected quantitative repeated measures data on psychological well-being during the lockdown via a set of four web-based surveys, which were administered intermittently to the same participants during two months of the COVID-19 lockdown in India. Online Google and Microsoft forms were circulated through social media platforms such as Facebook and LinkedIn to recruit a diverse pool of participants. This method of recruitment was suitable due to the restrictions on in-person interactions with strangers during the lockdown in India, and efficient, given the ability to recruit a diverse sample of participants very quickly. In addition to posting the forms on social media, they were circulated among the social networks of the authors. To increase the size and diversity of the sample, we requested the participants to share the forms among their peers. All the forms included a brief introduction describing the primary objectives of the study. Additionally, the participants were informed that their participation was completely voluntary.

We deployed our first survey on March 29th, 2020, during the first week of the lockdown. The online survey was open for two weeks. We received responses from 793 participants in this round (T1). However, only 561 of them shared their interest in participating in the subsequent surveys. We rolled out the second follow-up survey on April 14th, the third on May 2nd, and the final follow-up survey on May 24th. Since we recruited our participants through social media, the second (T2), third (T3), and the fourth (T4) surveys received responses from new participants as well. Our analytical sample for the current study included only the 159 participants from India who voluntarily participated in all four rounds of surveys. However, we measured the outcomes of interest in the present study only at two-time points. Data on anxiety and stress were collected during T1 and T4, while we measured depressive symptoms during T2 and T4.

### Response variables

#### Anxiety

We used the Generalized Anxiety Disorder-7 (GAD-7) scale to assess anxiety. The scale is used widely with a demonstrated high reliability and validity [28]. The scale included seven items such as “*Feeling nervous*, *anxious or on edge; Not being able to stop or control worrying*.” The responses were recorded on a 4-point Likert scale ranging from *never* (0) to *nearly every day* (3). The total score of GAD-7 ranged from 0 to 21, where greater score predicted higher levels of anxiety [28]. The scale has been previously used in the Indian context [29, 30]. Anxiety was measured at time points T1 and T4. We found a strong internal consistency in our sample with a Cronbach’s alpha of 0.85 at T1. and 0.91 at T4.

#### Stress

The single item, *“Stress means a situation in which a person feels tense*, *restless*, *nervous or anxious or is unable to sleep at night because his/her mind is troubled all the time*. *Do you feel this kind of stress these days*? [31] was used to measure the level of stress experienced by the participants. The participants responded on a 5-point Likert ranging from *not at all* (1) to *very much* (5). We measured stress at T1 and T4.

#### Depressive symptoms

We used two items on depressive symptoms from the Patient Health Questionnaire-4 (PHQ-4) developed and validated by Kroenke et al. [32] to assess depressive symptoms. The scale included items such as *“Over the last two weeks*, *how often have you been bothered by the following*: *Feeling down*, *depressed or hopeless; Little interest or pleasure in doing things”* Responses were recorded on a 4-point Likert scale ranging from *not at all* (0) to *nearly every day* (3). A previous study has used this scale in the Indian population [33]. We collected data on depressive symptoms at T2 and T4. The PHQ-4 scale had good internal consistency in our sample with a Cronbach’s alpha of 0.77 at T2 and at T4 was 0.81. The length of our survey restricted the use of longer scales. Several reports from our pretest suggested that participants found the longer surveys tiresome.

At time T1 (March 29th, 2020), we found moderate to small correlations of the scores of depressive symptoms with that of anxiety and stress (correlation coefficient: 0.60 and 0.49, respectively) in our sample. On the other hand, the correlation between the scores of stress and anxiety was strong (correlation coefficient: 0.76) at T1. At time T4 (May 24th, 2020), we found a moderate correlation between the scores of anxiety and stress (correlation coefficient: 0.59).

### Predictors

#### Sociodemographic characteristics

Sociodemographic information of the participants included age (in years), gender (male/female/non-binary), education (high school or less/some college/above college), annual income (in Indian Rupees) (0–3,00,000 (low)/ 3,00,000–7,00,000 (medium)/ 7,00,000 and above (high)), and place of residence (rural/ urban). To reduce the length of the survey, we included a few sociodemographic variables in each of the four surveys.

#### History of mental health

We used the question, “*Have you suffered from depression or any mental health issues before*” to indicate if the participant had any history of mental illness. The responses to this question were recorded as *yes* (1) or *no* (0).

#### Social support

We used the following two items to measure social support: “*Is there someone you could count on to help you if you contracted the virus and got sick*, *for example*, *to take you to the doctor or help you with daily chores*?*”*, and “*If in these times due to unforeseen circumstances you need some extra help financially*, *could you count on anyone to help you*, *for example*, *by paying any bills*, *housing costs*, *medical expenses*, *or providing you with food or clothes*?*”* Responses were converted to *yes* (1) or *no* (0).

#### Resilience

To assess resilience, we used the two-item brief Connor-Davidson Resilience Scale, developed by Vaishnavi, Connor & Davidson (2007) [34]. It includes items such as *“Are you someone who is*: *Able to adapt to change; Tend to bounce back after illness or hardship*?*”* The participants responded on a 5-point Likert scale ranging from strongly disagree (1) to strongly agree (5). We collected data on resilience at T1. The resilience scale (RISC-2) showed low internal consistency at T1 with a Cronbach’s alpha of 0.60.

### Other variables

#### Responsibility

We used self-reports at T2 to assess whether there was an increase in responsibilities (social, financial, household, and personal) of the participants during the lockdown. The responses were recorded as *yes* (1) or *no* (0). The aggregate of the four responsibility scores reflected the total increased responsibility score.

### Statistical analyses

We first performed descriptive analyses to compute the distribution of outcomes at different time points across gender, relationship status, education, annual income, and place of residence. Next, we fitted separate linear two-level (observations nested within individuals) multilevel models for each outcome (anxiety, stress, and depressive symptoms) to assess the temporal changes in the outcomes. These models accounted for any autocorrelation of the responses from the same participants. Model 1 included the primary predictor time and the sociodemographic variables. Model 2 additionally adjusted for the interaction of gender with time. Model 3 further adjusted for the buffer factors (social support and resilience) and a history of mental health issues. Additionally, we ran an ANOVA model to analyze the gender differences in responsibilities during the lockdown. We set alpha at 0.05 in our study. All our models were run in STATA version 12 [35].

### Ethical considerations

The study was approved by the Institutional Ethics Committee, Indian Institute of Technology, Gandhinagar. All participants were informed about their voluntary participation through the introduction section in the Google and Microsoft forms. The participants were requested to carefully read the instructions and the details about the study, and then respond to the survey. The participants were also informed that the collected data would be kept confidential and not shared with anyone outside the research team. Email addresses of participants who gave consent for follow-up were collected as identifying information. Statistical analyses were performed on de-identified data.

## Results

### Preliminary results

We collected data on psychological outcomes from 159 Indian adults across a period of two months during the lockdown. Our sample comprised relatively young participants (mean age = 27.44 years, SD = 9.17 years). About 65% of the sample were men and the remaining were women. The annual income of nearly half of the participants was below 3,00,000 Indian Rupees (a cut-off representing an income allowing decent living in a one-bedroom apartment for a couple in most urban areas of India). About 55% of the participants were at least college-educated, while only about 11% reported having an educational qualification less than high school. The distribution of the psychological outcomes across these groups is presented in Table 1.

**Table 1:** The distribution (mean, standard deviation) of anxiety, stress, and depressive symptoms at times T1, T2, and T4 across gender, annual income, education, and place of residence (N = 159).

	Anxiety		Stress		Depressive symptoms	
	T1	T4	T1	T4	T2	T4
**Gender**						
Men	4.02 (4.41)	4.17 (4.19)	1.98 (0.90)	2.29 (1.07)	1.42 (1.27)	1.67 (1.58)
Women	4.61 (4.14)	6.72 (4.83)	2.36 (1.13)	3.25 (1.22)	1.59 (1.41)	2.19 (1.58)
**Annual income**						
Low	4.59 (4.45)	5.44 (4.94)	2.10 (1.03)	2.71 (1.20)	1.57 (1.23)	1.99 (1.62)
Medium	4.37 (4.41)	5.15 (4.59)	2.18 (1.05)	2.69 (1.36)	1.64 (1.68)	2.15 (1.81)
High	3.30 (3.85)	4.08 (3.57)	2.08 (0.91)	2.37 (1.05)	1.31 (1.02)	1.24 (1.10)
**Education**						
High school or less	5.39 (5.86)	4.17 (4.66)	1.89 (0.76)	2.78 (1.32)	1.28 (0.96)	1.5 (1.25)
Some college	3.96 (4.12)	6.02 (5.18)	1.96 (0.90)	2.77 (1.28)	1.69 (1.35)	2.35 (1.70)
Above college	4.14 (4.05)	4.63 (4.09)	2.25 (1.09)	2.60 (1.14)	1.40 (1.36)	1.62 (1.53)
**Place of residence**						
Rural	4.52 (3.65)	6.38 (5.13)	2.21 (0.98)	2.75 (1.33)	1.71 (0.95)	1.96 (1.57)
Urban	4.18 (4.42)	4.81 (4.44)	2.09 (1.00)	2.6 (1.19)	1.44 (1.37)	1.83 (1.60)

T1: Timepoint 1- March 29^th^, 2020

T2: Timepoint 2- April 14^th^, 2020

T4: Timepoint 4- May 24^th^, 2020

Following the cut-offs suggested by Spitzer et al. [28], 70.8% of the participants reported being mildly anxious, 18.8% stated being moderately anxious, and 10.4% reported severe anxiety symptoms at T1 (early days of the lockdown). At T4 (after eight weeks of lockdown), the prevalence of moderate anxiety and severe anxiety increased to 26% and 12.7%, respectively. Similarly, the prevalence of intermediate stress and high stress [36] increased from 20.1% to 27% and 10.7% to 24%, respectively. Our results also showed that while 14.8% of the participants reported being depressed [32, 37] at T2, the prevalence increased to 26.1% during T4.

### Trends in anxiety, stress, and depressive symptoms during the lockdown

Our multilevel models, adjusted for sociodemographic variables showed an increase in anxiety (**β** = 0.81, CI: 0.03, 1.60) (Table 2) and stress scores (**β** = 0.51, CI: 0.32, 0.70) (Table 3) during the two months (T1 to T4) of follow-up. We also found an increase in depressive symptoms (**β** = 0.37, CI: 0.13, 0.60) between T2 and T4, independent of the covariates (Table 4). Anxiety (**β** = 1.62, CI: 0.44, 2.81) and stress scores (**β** = 0.65, CI: 0.37, 0.94) were found to be higher among women, versus men, even after accounting for time and sociodemographic factors. However, we could not find statistically significant associations of the other sociodemographic variables (age, annual income, educational qualification, and place of residence) with the psychological outcomes.

**Table 2:** Changes (regression coefficients, 95% CI) in anxiety between week 1 and week 8 of the lockdown in India (N = 159).

	Model 1	Model 2	Model 3
**Time**	0.814 (0.025, 1.602)	0.125 (-0.831, 1.082)	0.168 (-0.860, 1.197)
**Gender (ref- Men)**			
Women	1.624 (0.443, 2.805)	0.619 (-0.820, 2.059)	0.306 (-1.170, 1.782)
Age	-0.042 (-0.113, 0.027)	-0.043 (-0.113, 0.027)	-0.055 (-0.125, 0.014)
**Annual income (ref-Low)**			
Medium	0.315 (-1.125, 1.756)	0.286 (-1.155, 1.727)	0.069 (-1.348, 1.486)
High	-0.734 (-2.178, 0.710)	-0.742 (-2.187, 0.702)	-0.694 (-2.145, 0.757)
**Education (ref- High school or less)**			
Some college	0.303 (-1.611, 2.217)	0.320 (-1.594, 2.236)	1.395 (-0.578, 3.370)
Above college	-0.399 (-2.393, 1.595)	-0.376 (-2.371, 1.618)	0.777 (-1.243, 2.799)
**Place of residence (ref- Rural)**			
Urban	-0.783 (-2.337, 0.769)	-0.790 (-2.343, 0.763)	-1.180 (-2.784, 0.423)
Gender*time		1.983 (0.357, 3.609)	1.893 (0.155, 3.631)
**Social support**			-0.520 (-1.574, 0.533)
**Resilience**			-0.515 (-0.896, -0.135)
**History of mental health (ref-No history)**			2.236 (0.799, 3.673)

Model 1: Adjusted for sociodemographic variables

Model 2: Includes interaction term (gender and time) adjusted for sociodemographic variables

Model 3: Additionally, adjusted for social support, resilience, and history of mental health

**Table 3:** Changes (regression coefficients, 95% CI) in stress between week 1 and week 8 of the lockdown in India (N = 159).

	Model 1	Model 2	Model 3
**Time**	0.509 (0.315, 0.703)	0.307 (0.073, 0.541)	0.322 (0.075, 0.569)
**Gender (ref- Men)**	0.654 (0.370, 0.938)	0.362 (0.016, 0.709)	0.270 (-0.092, 0.632)
**Age**	-0.009 (-0.025, 0.006)	-0.009 (-0.025, 0.006)	-0.011 (-0.027, 0.005)
**Annual income (ref- Low)**			
Medium	0.081 (-0.264, 0.428)	0.081 (-0.264, 0.428)	0.074 (-0.280, 0.429)
High	-0.083 (-0.428, 0.262)	-0.083 (-0.428, 0.262)	-0.056 (-0.418, 0.305)
**Education (ref- High school or less)**			
Some college	0.290 (-0.172, 0.752)	0.290 (-0.172, 0.752)	0.398 (-0.098, 0.896)
Above college	0.282 (-0.196, 0.761)	0.282 (-0.196, 0.761)	0.375 (-0.131, 0.882)
**Place of residence (ref- rural)**			
Urban	-0.062 (-0.435, 0.310)	-0.062 (-0.435, 0.310)	-0.213 (-0.613, 0.186)
Gender* time		0.583 (0.185, 0.980)	0.577 (0.160, 0.994)
**Social support**			0.025 (-0.238, 0.288)
**Resilience**			-0.063 (-0.158, 0.031)
**History of mental health (ref-No history)**			0.439 (0.085, 0.794)

Model 1: Adjusted for sociodemographic variables

Model 2: Includes interaction term (gender and time) adjusted for sociodemographic variables

Model 3: Additionally, adjusted for social support, resilience, and history of mental health

**Table 4:** Changes (regression coefficients, 95% CI) in depressive symptoms between week 3 and week 8 of the lockdown in India (N = 159).

	Model 1	Model 2	Model 3
**Time**	0.367 (0.130, 0.604)	0.248 (-0.042, 0.539)	0.181 (-0.122, 0.484)
**Gender (ref- Men)**	0.380 (-0.011, 0.773)	0.208 (-0.255, 0.672)	0.055 (-0.439, 0.550)
**Age**	-0.021 (-0.043, 0.000)	-0.021 (-0.043, 0.000)	-0.028 (-0.052, -0.005)
**Annual income (ref- Low)**			
Medium	0.325 (-0.152, 0.804)	0.325 (-0.153, 0.803)	0.428 (-0.078, 0.934)
High	-0.369 (-0.847, 0.107)	-0.370 (-0.847, 0.107)	-0.286 (-0.802, 0.229)
**Education (ref- High school or less)**			
Some college	0.659 (0.021, 1.298)	0.660 (0.022, 1.298)	0.598 (-0.110, 1.307)
Above college	0.165 (-0.494, 0.825)	0.166 (-0.493, 0.826)	0.163 (-0.558, 0.884)
**Place of residence (ref- rural)**			
Urban	-0.062 (-0.577, 0.451)	-0.062 (-0.576, 0.452)	-0.082 (-0.652, 0.487)
Gender*time		0.344 (-0.151, 0.839)	0.369 (-0.142, 0.882)
**Social support**			-0.112 (-0.488, 0.264)
**Resilience**			-0.004 (-0.140, 0.130)
**History of mental health (ref-No history)**			0.705 (0.200, 1.209)

Model 1: Adjusted for sociodemographic variables

Model 2: Includes interaction term (gender and time) adjusted for sociodemographic variables

Model 3: Additionally, adjusted for social support, resilience, and history of mental health

### Differential increase of anxiety, stress, and depressive symptoms across gender

The interaction of gender with time was statistically significant for the anxiety (p-value for interaction = 0.017) and stress (p-value for interaction = 0.004) outcomes in models, adjusted for sociodemographic variables. Women showed a greater rate of increase in anxiety and stress scores between T1 and T4, as compared to men, after accounting for the sociodemographic covariates. Keeping age, education, income, and place of residence constant, men showed an average increase of 0.13 points in anxiety and 0.31 points in stress scores during the follow-up. The corresponding figures for women were 2.73 and 1.25 points, respectively. Further, this interaction was found to be significant even after adjusting for social support, resilience, and a history of mental health.

However, we did not find statistical evidence supporting the interaction of gender with time in our models for depressive symptoms.

### Other findings

We found statistically significant and positive associations of a history of mental health issues with anxiety (**β** = 2.24, CI: 0.80, 3.67), stress (**β** = 0.44, CI: 0.09, 0.79), and depressive symptoms (**β** = 0.71, CI: 0.20, 1.21), independent of all covariates.

Our fully adjusted models also found that a higher level of resilience was associated with lower anxiety, stress, and depressive symptoms. However, the associations of social support with the psychological outcomes were not statistically significant in our models.

Further, one-way ANOVA analysis highlighted a gendered difference in reports of increased responsibilities during the lockdown (p<0.001). Our results showed that women (M = 1.35, SD = 0.97) reported a greater increase in their responsibilities compared to men (M = 0.90, SD = 1.04) during the lockdown.

## Discussion

Using repeated measures of psychological outcomes from 159 Indian adults during two months of the COVID-19 lockdown, we found that there were statistically significant increases in stress, anxiety, and depressive symptoms over this period. Moreover, this increase in adverse psychological outcomes was found to be greater among women compared to men. We also found that a higher level of an individual’s resilience was related to lower levels of anxiety, stress, and depressive symptoms.

Our findings suggest that anxiety, stress, and depressive symptoms increased during the lockdown among adults in India. While depressive symptoms increased in both the genders, the effect size was modest. Nevertheless, the increase in the adverse psychological outcomes could be because of several reasons. First, the nationwide lockdown disrupted the professional, personal, and social lives of individuals, which potentially impacted their psychological well-being. Moreover, the periodic extensions of the lockdown over a considerably long period, accompanied by a steep increase in the number of COVID-19 cases in the country, and even worldwide, probably worsened their anxiety, stress, and depressive symptoms over time. Each announcement of the extension of the lockdown might have elevated the levels of anxiety by engendering a perception of unpredictability. Further, the initial shocks of the lockdown followed by the social isolation maintained for a prolonged time, combined with the emotional and financial losses incurred during the lockdown, might have created a synergistic psychological impact. Notably, the Government of India announced a relaxation of restrictions on certain activities even while extending the lockdown. These relaxations included the resumption of trains, opening of small shops, and inter-state mobility. However, these graded “unlocks” (which potentially allowed increasing physical mobility as well) were not accompanied by reports of a reduction in the number of new COVID-19 cases in India. On the contrary, the case numbers shot up just as the “unlocking” began, which could have reduced the confidence of the citizens, leading to a higher perceived risk of contracting the disease and further increasing their stress, anxiety, and depressive symptoms. However, these findings of our study are contrary to those found by Wang et al. [38] in China, where stress and anxiety were found to be stable across 4 weeks of lockdown. The explanation provided by Wang et al. highlights that China recorded substantial improvements in curbing the spread of the virus due to their rapid decisive measures and the greater number of recovered patients. This might have instilled greater confidence in their public health measures among the Chinese, thus avoiding a worsening of the psychological toll of a prolonged lockdown [38].

As per the findings of our study, compared to men, women had a greater increase in stress, anxiety, and depressive symptoms during the lockdown. Notably, the increases in anxiety and stress in our sample were primarily due to this evidence of a greater increase in anxiety and stress observed in women. There are two potential explanations for this finding. First, it was found that women in the Indian context have significantly more household responsibilities during the lockdown primarily because of the skewed gendered division of household labor in India [39, 40]. For instance, due to the closure of schools and offices, all family members could be staying indoors, leading to an increase in household burden for women who are expected to shoulder most of the childcare, cooking, cleaning, and other household management. This would leave them with very limited time for themselves. Corroborating this, Viglione [41] reported lower publication rates among female academicians in North America compared to their male counterparts during this pandemic, across all disciplines. Our results from India, a society with stronger patriarchy, supports this narrative. We found that women reported a greater increase in their responsibilities during the lockdown compared to men. These added responsibilities, combined with the lack of time for themselves, could increase their stress, anxiety, and depression levels much more than the increase among men. Second, social isolation and the restrictions on physical mobility might increase the exposure of women to hostility at home, especially among women who were already vulnerable to domestic violence. Previous studies have reported an increase in the risk of women across the world experiencing hostility during the lockdown period [42–44]. The high prevalence (~30%) of domestic violence [19] in India is a reflection of the vulnerability of Indian women to domestic violence (ranging from emotional abuse from family members to physical/sexual abuse from intimate partners). Any risk of such hostility could worsen during the lockdown. Prolonged exposure to any risk of domestic hostility (emotional, physical, or sexual) could lead to an increase in stress, anxiety, and depression among Indian women during the lockdown.

We found that the greater increase in stress and anxiety among women versus men persisted even after accounting for social support and resilience. This suggests that this gendered pattern was strong enough to persist despite any protective effect exerted by these buffering factors. Even though the interaction of depressive symptoms with gender was not statistically significant, it suggested that the rate of increase in depressive symptoms was higher among women than men, thus fitting the pattern observed with anxiety and stress by Ozamiz-Etxebarria et al. [27] in Northern Spain. They found higher levels of anxiety, stress, and depression among men compared to women. This contrast with our results could be because of the cultural differences between the two countries with regard to the gendered division of household labor [45, 46].

We also found that persons with a history of mental health issues were likely to report an increase in anxiety, stress, and depressive symptoms during the period of lockdown. Previous research has highlighted that situations of social avoidance could cause a relapse of trauma and depressive events [47]. The social isolation, added responsibilities, and any lack of perceived social support (due to physical mobility restrictions) could trigger those with past depressive episodes. Studies have shown higher stress and anxiety during the lockdown in India [48].

It is important to implement preventive and therapeutic interventions to address the adverse effects of the pandemic, especially the lockdown. Interventions such as cognitive behavior therapy (CBT) and mindfulness-based therapies (MBTs) could be useful in treating psychiatric symptoms during COVID-19 [25]. A Chinese study highlighted a lower prevalence of psychiatric symptoms after the lockdown because of the psychoneuroimmunity prevention measures administered by the Chinese government, which helped instill a sense of confidence in the people [49]. Many such measures suggested by Tan et al. [49] were also adopted in India. For instance, the Ministry of Family Health and Welfare [50] has widely spread public service messages advising all to wear masks, adopt hygiene practices, and social distancing while also promoting precautionary measures at workplaces.

### Limitations and strengths

The study has several limitations, which we acknowledge. First, the survey was conducted online, limiting the sample to only those who had access to the Internet. However, the online method of recruitment helped us collect data from a diverse sample within a short time, given the restriction of physical mobility due to the lockdown. Second, we could not follow-up with the majority of our participants during the study. This was likely because we relied on only one mode of communication, their email, for follow-up. In the chaos of the COVID-19 pandemic and the challenges it brought, the participants might have missed the emails related to the follow-ups. However, to our knowledge, this is the first web-based longitudinal study from India capturing key insights of psychological well-being *over time* during a lockdown that was periodically extended. Third, our sample size of 159 participants was modest; yet it allowed us to analyze the changes in the psychological outcomes during the lockdown with several statistically significant results. Fourth, since the survey was in English, all participants who volunteered were comfortable in English, and unsurprisingly, 89% had some college education. Therefore, the results cannot be generalized to the whole of India. However, we found policy-relevant results showing an increase of anxiety, stress, and depressive symptoms in a relatively well-educated sample. We argue that the relatively underprivileged (socially as well as economically) are potentially even more vulnerable to such adverse psychological outcomes during the lockdown. Another major limitation that the readers need to bear in mind while interpreting the results is our use of a single-item scale to assess stress in our sample. While we found strong internal consistency for the 2-item short scale we used to measure depressive symptoms, the nature of the one-item stress scale did not allow us to assess internal consistency to measure reliability. Further, such single-item scales are more vulnerable to random measurement errors, which could be minimized by using longer scales. For instance, our single-item stress scale could create ambiguity among the respondents while interpreting the item, leading to further biases [51]. All of these weaken our ability to accurately measure stress. Despite using this crude measure, we were able to detect important patterns in our analysis. While we acknowledge this limitation, we preferred using these shorter scales because the administration of shorter scales is advised in vulnerable populations to avoid increasing their psychological toll [52]. Our longitudinal approach required the repeated administration of the scales during the lockdown. We believed that asking participants to respond to longer scales during a stressful time, such as the lockdown, could overburden them. Therefore, we chose to minimize the risk of respondent burden by carefully balancing the number of questions assessing various psychological constructs. Thus, we employed widely used shorter scales to measure stress and depressive symptoms. We also caution that the internal consistency of the RISC scale was low at 0.60. Lastly, we used self-reported measures to assess anxiety, stress, and depressive symptoms in our sample. While clinical interviews would have yielded better results, we argue that the use of validated, reliable, and widely cited scales to measure anxiety and depressive symptoms make our results credible.

Despite these limitations, the strength of our study lies in its longitudinal nature, which sheds light on the trend of psychological outcomes during the lockdown in India. Moreover, we measured the outcomes at two interesting time points, one during the initiation of the lockdown and the other during a phase of relative relaxation, allowing us to assess if the psychological outcomes changed during differing dynamics of the lockdown. Despite a modest sample size, we also found statistical evidence to highlight the gender-based disparities in the effect of the lockdown, which was likely due to the gendered interpretation of circumstances created due to this pandemic plus a gendered emotional and behavioral response to the subsequent lockdown, all of which could, in turn, be socially determined.

### Implications

Our salient findings highlight a long-term impact of the lockdown on the mental well-being of Indian adults. These findings can help mental health policymakers to design disaster-response policies to address the psychological needs of the citizens during such crises, including a plan for follow-ups. Additionally, our findings highlight the need for these policies to be socially inclusive, with prioritized care for the vulnerable such as women and those with existing mental health issues. A long-term perspective on preparedness would benefit from policies designed to enhance resilience among Indian citizens and prepare them to adapt to such crises.

An immediate response to our findings would be the involvement of philanthropic non-governmental organizations, social workers, and other community service providers to provide emotional support to communities during and after the COVID-19 pandemic, with a special focus on women and the underprivileged.

## Conclusions

The COVID-19 crisis and the accompanying lockdown have undoubtedly affected every individual in one way or the other. While the lockdown may help in effectively addressing this pandemic, the state and society at large need to be sensitive to the mental health impacts of a long-drawn-out lockdown. Vulnerable populations such as women and the marginalized deserve immediate attention. However, it behooves society to also address the root causes driving the unequal distribution of psychological distress during such crises.
